# Microbial community and flavor analyses of fermented grains of Furou-type Baijiu

**DOI:** 10.3389/fmicb.2025.1628609

**Published:** 2025-12-08

**Authors:** Yanyan Tang, Yanbo Liu, Pengpeng Zhang, Hongliang Liu, Yonghua Wang, Huawei Li, Jinxiao Zhu, Chunmei Pan

**Affiliations:** 1 College of Food and Biological Engineering (Liquor College), Henan University of Animal Husbandry and Economy, Zhengzhou, China; 2 School of Life and Health Sciences, Hubei University of Technology, Wuhan, China; 3 Henan Province Engineering Technology Research Center of Liquor Style, Henan University of Animal Husbandry and Economy, Zhengzhou, China; 4 Henan Province Brewing Special Grain Development and Application Engineering Research Center, Henan University of Animal Husbandry and Economy, Zhengzhou, China; 5 Zhengzhou Key Laboratory of Liquor Brewing Microbial Technology, Henan University of Animal Husbandry and Economy, Zhengzhou, China; 6 College of Biological Engineering, Henan University of Technology, Zhengzhou, China; 7 Henan Caihongfang Distillery Co., Ltd., Xincai, China

**Keywords:** Furou-type Baijiu, fermented grains, microorganisms, SourceTracker, GC–MS

## Abstract

**Introduction:**

In this study, we used single-molecule real-time sequencing to investigate the microbial community structure of jiuqu, pit mud, and fermented grains, before and after cellaring, in the brewing environment.

**Methods:**

We detected the volatile flavor substances in the fermented grains using gas chromatography–mass spectrometry (GC–MS), and examined the changes in microbial community structure, before and after cellaring, as well as the correlation between flavor and microorganisms in Furou-type Baijiu. At the same time, the microbial traceability technique was used to investigate the contribution of fermented grains to the brewing microbiota, before and after cellaring.

**Results and discussion:**

The results showed that the microbial diversity and richness of the fermented grains were higher than those at the time of cellaring. The dominant bacterial species changed from *Bacillus subtilis* to *Acetilactobacillus jinshanensis*, and the dominant fungal genus, *Saccharomyces* sp., gradually increased. The relative abundance of *Wickerhamomyces anomalus* gradually decreased. The contents of acids, alcohols, and esters, before and after cellaring, were primarily influenced by the microbial community structure, while the relative amounts of flavor substances in fermented grains were significantly higher than those in stacked samples. The traceability analysis revealed that the microbial bacterial community in the fermented grains, both before and after entering the cellar, mainly originated from Fuqu and pit mud, while the fungal community primarily originated from Fuqu. This study offers insights into regulating of microbial community diversity to produce high-quality Furou-type Baijiu.

## 1 Introduction

China Baijiu holds a pivotal position in the development of China’s Baijiu (Wei et al., 2020). The types and proportions of microorganisms vary due to differences in geographic climate and production processes. It is the metabolic activity of the different types and proportions of microorganisms during the fermentation process that produces the various volatile compounds that result in the production of different flavors of Baijiu (Gou et al., 2015; Tan et al., 2019). Furou-type Baijiu is a natural and appropriate expression of the fusion of the four types of Baijiu aroma, thick, saucy, clear, sesame aroma, thick does not lose the sauce, the sauce does not pressure the dense, the aroma of the harmonic and natural, give people a more comfortable drinking experience, the taste is full, sweet, long aftertaste, the ripeness of the body, the sheer sweetness of the Baijiu, the style is unique, with a typical flavor characteristics of the Central Plains of the Baijiu flavor features and stylistic characteristics.

The solid-state fermentation of Baijiu is an open process, in which a variety of microorganisms are involved in the formation of a typical “multi-microbial co-fermentation” phenomenon, in which these microorganisms interact with each other through competition, symbiosis, or other forms of mutual benefit, and ultimately form a complex microbial community structure (Tong et al., 2023; Xu et al., 2017). In the Baijiu production process, the open environment is used for steps such as steaming and cooling the grains, as well as adding daqu. This process inevitably introduces a multitude microorganisms from the air, soil, and tools used (Guiming et al., 2021; Lu et al., 2017). These microorganisms from the environment are introduced into fermented grains in different ways, which can enhance the microbial population in the grains to a certain extent, thus enriching the functional flora and improving the fermentation efficiency (Jin et al., 2017; Pang et al., 2018; Shi et al., 2011; Wang, 2022). Stacking fermentation is an intermediate operation between daqu fermentation and cellar fermentation. It enriches the microecology of Baijiu through enrichment and expansion, playing a crucial role in the yield and quality of Baijiu (Chen et al., 2024). The multiple microbial sources of daqu, pit mud, sorghum, air, and ground during the Baijiu brewing process influence the microbial composition of fermented grains through different channels (Wang, 2022; Wang et al., 2016), Bacteria, saccharomycete and molds produce an abundance of flavor substances and their precursors through the action of different enzymes during the fermentation process(Li et al., 2023; You et al., 2021). The characteristics of these microorganisms thus indirectly affect the flavor and quality of the final product. Tracing the origin of these microorganisms has become an important task in the field of Baijiu research. With the development of microbiomics technology, single-molecule real-time (PacBio SMRT) sequencing has been widely used in brewing microbial diversity studies. Compared to second-generation sequencing, PacBio SMRT sequencing provides ultra-long read lengths, the detection of genomic modifications without amplification. It is fast, real-time, and suitable for species-level analysis of microbial communities (Hestand and Ameur, 2019).

Currently, microbial traceability studies primarily use microbiome technology combined with statistical methods to analyze and investigate. Previously, researchers used high-throughput sequencing combined with SourceTracker analysis to explore the microbial communities in fermented grains of soy sauce-flavored Baijiu and the sources of microbial communities contributed by volatile components. They found that the environmental microorganisms in the tools were the primary sources of bacterial and fungal communities in the stacked fermentation. The environmental microorganisms in tools were found to be the primary source of bacterial and fungal communities in stacked fermentation; at the end of fermentation in the cellar, the pit mud was the primary environmental contributor to the bacterial community in the fermented grains, and the tools, the daqu and the cellar mash were the main contributors to the fungal community (Lu et al., 2022). A variety of microorganisms in the daqu and production environment are involved in the Baijiu fermentation process, playing a crucial role in the formation of flavors (Hu et al., 2021; Liu et al., 2017).

Microbial traceability studies on fermented grains have primarily focused on the genus level, with fewer studies at the species level. There are fewer reports on the traceability of microorganisms of Furou-type Baijiu at the species level. Therefore, in this study, we used SMRT combined with SourceTracker analysis to investigate the microbial community structure of daqu, pit sludge, and fermented grains, before and after cellaring, as well as the brewing environment. Additionally, we detected the volatile flavor substances in the fermented grains, before and after cellaring, by GC–MS and investigated the influence of the environmental microorganisms on the microbial community of those grains. Finally, we analyzed the effect of the environmental microorganisms on the microbial community of fermented grains, before and after cellaring, of Furou-type Baijiu microbial community and analyze the correlation between flavor and microorganisms. This study not only helps to understand the microbial migration pattern in Baijiu production but also reveals the role of microorganisms and their uniqueness in the flavor formation of Furou-type Baijiu, which is of great significance for stabilizing and improving the Baijiu production process.

## 2 Materials and methods

### 2.1 Sample collection

All samples were taken from Henan Caihongfang Distillery Co., Ltd. Using premoistened and washed 0.1 mol/L phosphate buffer solution, sterile degreased cotton balls, and random wipes, samples were taken on the workshop floor, and tools were sampled in triplicate for Tools (GJ). High-temperature daqu and fuqu used for production were sampled randomly at five locations in the same daqu room, crushed, and then daqu and fuqu were sampled in triplicate for Daqu (GW) and Fuqu (FQ) samples. The 5-point stratified blending method was used to take the heap environment, the heap 40 h samples and fermented grains in triplicate as heap environment (DH), heap 40 h samples (DS) and fermented grains (JP), and the 5-point pit mud blending from the cellar wall around the different layers of the cellar and the bottom layer as pit mud (JN), for a total of 21 samples (Supplementary Figure S2), and all the samples were collected into a sterile plastic bag and transported to the laboratory −80°C refrigerator by placing them immediately on dry ice for sequencing analyses and flavor assays. The same experimental procedure was performed 3 times independently under the same experimental conditions.

### 2.2 DNA extraction and PCR amplification

To obtain microbial community genomic DNA, the samples were processed using the E. Z. N. A. Soil DNA Kit (Omega Bio-Tek, USA). The extracted DNA was examined on 1% agarose gels, and its concentration and purity were determined using a NanoDrop2000 spectrophotometer (Thermo Scientific, USA). For bacterial communities, the full-length region of the bacterial 16S ribosomal RNA (rRNA) gene was amplified using universal bacterial primers 27F (5′-AGRGTTYGATYGATYGATYMTGGGCTCAG-3′) and 1492R (5′-RGYTACCTTGTTACGACTT-3′). For the internal transcribed spacer (ITS) region of fungi, the ITS full-length region sequence was amplified using primers ITS1F (5’-CTTGGTCATTTAGAGAGGAAGTAA-3′) and ITS4R (5’-TCCTCCGCTTATTGATATGC-3′). Primers were trailed with PacBio barcode sequences to distinguish each sample. The amplification reaction (20 μL volume) consisted of 4 μL of 5 × FastPfu buffer; 2.5 mM deoxynucleoside triphosphate (dNTP) 2 μL; 0.8 μL of forward primer (5 μM); 0.8 μL of reverse primer (5 μM); 0.4 μL of FastPfu DNA polymerase; 10 ng of template DNA; and DNase-free water. Polymerase chain reaction (PCR) amplification was performed as follows: initial denaturation at 95°C for 3 min, followed by denaturation at 95°C for 30 s, annealing at 60°C for 30 s, extension at 72°C for 45 s, and a single extension at 72°C for 10 min, ending at 4°C (ABI GeneAmp® Model 9,700, USA). Three PCR replicates were performed per sample, and the PCR products from the three replicates were then mixed and detected by electrophoresis on a 2% agarose gel. PCR products were purified using the AxyPrep DNA Gel Extraction Kit and evidenced using Qubit 4.0 (Thermo Fisher Scientific, USA).

### 2.3 DNA library construction and sequencing

DNA libraries were constructed using the SMRTbell Prep Kit 3.0 (Pacific Biosciences, CA, USA) according to the manufacturer’s (PacBio’s) instructions. Purified SMRT Bell libraries were sequenced by sequencing through the PacBio Sequel IIe System (Shanghai Meiji Biomedical Technology Co., Ltd.) in circular consensus sequencing (CCS) mode at SMRT-Link v11.0 to generate HiFi reads from sequenced subreads, and HiFi reads were barcode-identified and length-filtered. Using UPARSE 7.1 (Edgar, 2013; Stackebrandt and Goebel, 1994), the sequence similarity level was 97%. The most abundant sequence in each operational taxonomic unit (OTU) was selected as the representative sequence. All assays were repeated 3 times, and the results were expressed as mean and standard deviation. Based on the OUT information, Mothur v1.30 was used to calculate the sparse curve and *α*-diversity index.

### 2.4 Analysis of volatile compounds

Sample preparation: The sample to be measured was mixed with saturated NaCl solution and tert-amyl alcohol as internal standard, and then sonicated for 30 min. The tip of the extracted fiber of solid-phase carboxen and polydimethylsiloxane CAR/PDMS [75 μm CAR/PDMS, carbon molecular sieve/poly(dimethylsilane)] was inserted into the silicone stopper of the headspace vial and inserted into the sample for headspace adsorption for 30 min. GC conditions: the chromatographic column was HP- FFAP (30 m × 0.32 mm × 0.25 μm); no shunt, flow rate of 1.21 mL/min; inlet temperature: 250°C; heating program: 40°C hold for 3 min, 5°C/min to 60°C without holding, 8°C/min to 230°C hold for 7 min; mass spectrometry (MS) conditions: the interface temperature of 220°C, the ionization mode is an electron ionization source with electron energy of 70 eV. The mass spectrometry (MS) conditions were as follows: the interface temperature was 220°C, the ionization mode was an electron ionization source with an electron energy of 70 eV, and the ionization temperature was 200°C. The compounds were quantitatively analyzed by calculating the percentage of the GC peak area (Boyang et al., 2022).

### 2.5 Data analysis

Each assay was performed 3 times, and the results were described as the mean and standard deviation. Statistically significant differences (*p* ≤ 0.05, Duncan’s test) between Shannon and Chao 1 metrics were analyzed using one-way analysis of variance (ANOVA) in OriginPro2022 (OriginLab Corporation, MA, USA). Principal coordinate analysis (PCoA) was used to assess the ecological distance of different samples based on bray_curtis distance at the species level. To analyze the relationship between microorganisms and flavor, we calculated Spearman’s rank correlations between volatile compounds and the relative abundance of the first 20 microorganisms in the Baijiu fermented grains during the brewing process using the “stats” package of the R software (v3.6.3), which were visualized using heat maps. Microbial sources in fermented grains were analyzed by SourceTracker (v0.9.8) (University of Colorado, Boulder, CO, USA), and microbial populations from tools, stacking environments, daqu, fuqu, and pit mud were used as sources, and the microbial populations in the spirits, before and after cellaring, of Furou-type Baijiu were Receiving end. Raw data were statistically analyzed by Excel 2021 (Microsoft Corporation, Redmond, WA, USA), and bar graphs were plotted by OriginPro2022 (OriginLab Corporation, MA, USA).

## 3 Results

### 3.1 Community composition of the environment, jiuqu, pit mud, and fermented grains

**Figure 1:**
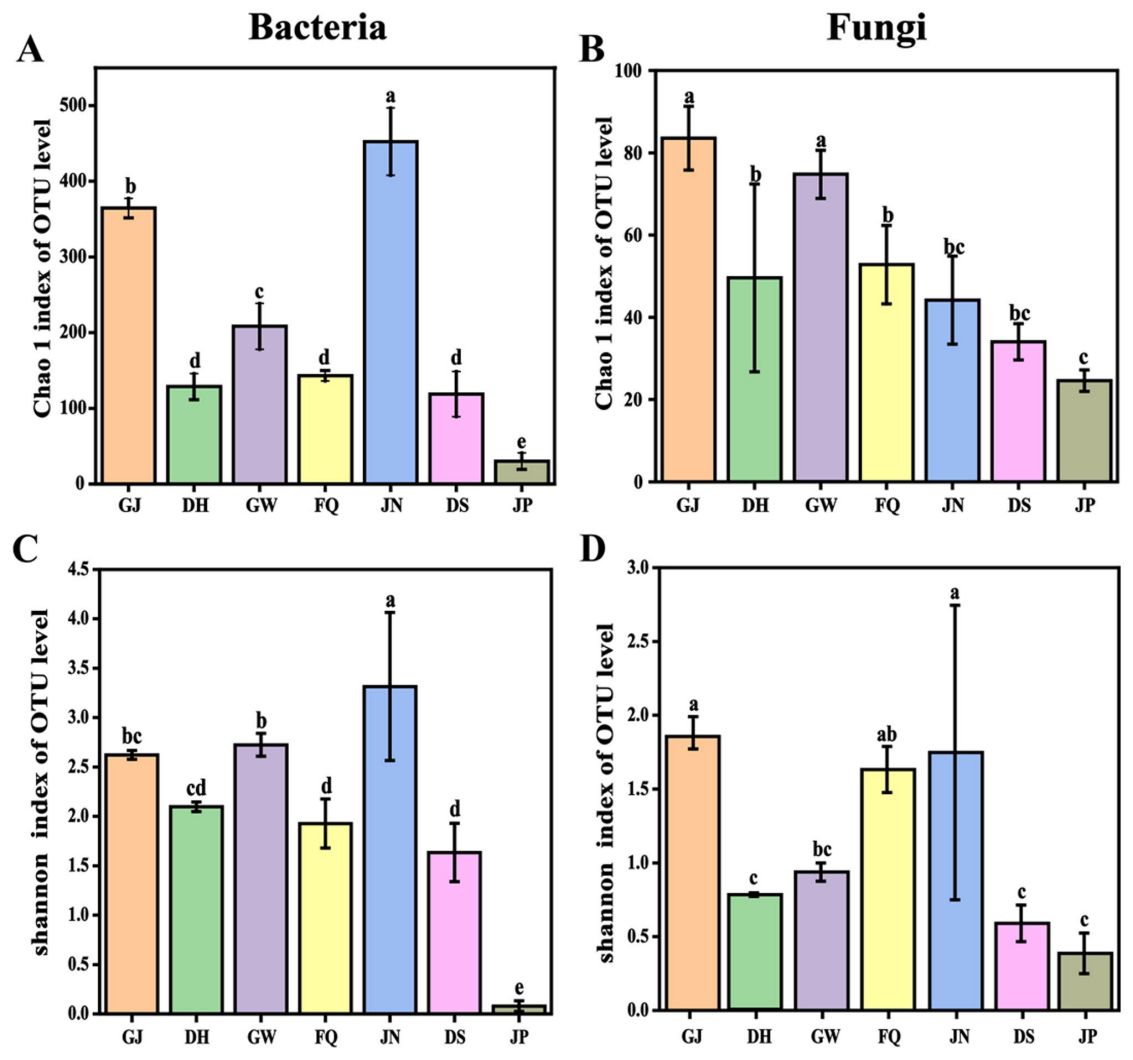
Alpha diversity of microbial communities in the environment, jiuqu, pit mud and fermented grains of Furou-type Baijiu before and after cellaring. Chao 1 index values for bacterial **(A)** and fungal communities **(B)**. Shannon index values of bacterial community **(C)** and fungal community **(D)**, *p* < 0.05.

The composition of environmental, jiuqu, pit mud, and fermented grains was analyzed by SMRT. A total of 967,466 valid sequence numbers from the 27F and 1,492 R regions of the bacterial rDNA genes were obtained in 21 samples, with read lengths ranging from 1,068 to 1,746 (Supplementary Table S1). The sparse curves showed a gradual flattening trend based on OTU counts, indicating that the current sequencing depth is sufficient to capture the vast majority of microbial phylotypes in the samples (Supplementary Figure S1). Alpha diversity, expressed as Shannon and Chao1 indices, reflects the diversity and richness of microbial communities (Lu et al., 2022), For fermented grain samples, before and after cellaring, bacterial community richness was significantly lower in JP than in DS (*p* < 0.05) (Figure 1A), and for ambient and daqu samples, bacterial community richness was significantly higher in JN and GJ than in FQ and GW (*p* < 0.05) (Figure 1A). Bacterial community diversity was significantly higher in JN, GW, and GJ than in FQ (*p* < 0.05) (Figure 1C). Among all samples, the bacterial community richness and diversity were lowest in JP (Figures 1A,C).

A total of 801,098 high-quality reads from the full-length region of the ITS for fungi were obtained in 21 samples, ranging from 357 to 873 read lengths (Supplementary Table S1). For fermented grain samples, before and after cellaring, fungal community richness was significantly lower in JP than DS (*p* < 0.05) (Figures 1B,D), and for ambient and daqu samples, fungal community richness was significantly higher in GJ and GW than in FQ, DH, and JN (*p* < 0.05) (Figure 1B). Fungal community diversity was significantly higher in GJ, JN, and FQ than in DH and GW (*p* < 0.05) (Figure 1D). Fungal community richness and diversity were lowest in JP among all samples (Figures 1B,D).

**Figure 2:**
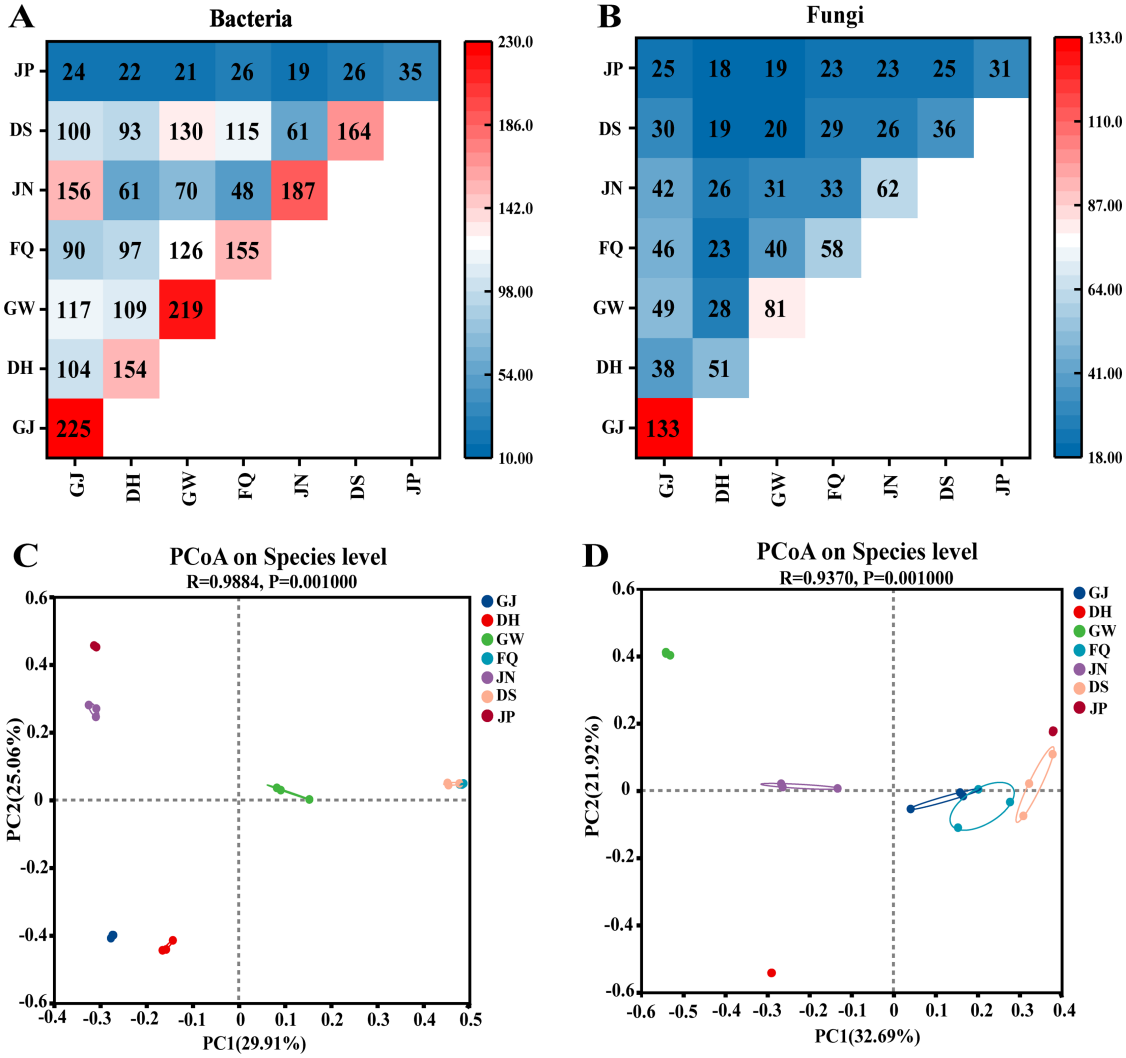
Sharing of microbial taxa between samples: **(A)**: shared bacterial taxa; **(B)**: shared fungal taxa; shared fungal taxa and Beta diversity assessed based on species-level bray_curtis principal coordinate analysis (PCoA) plots [**(C)**: bacteria; **(D)**: fungi] *p* < 0.05.

Further taxonomic annotation of the microbial OUT sequences at the species level revealed that a total of 383 bacterial species were identified across all samples. Significant changes occurred in DS and JP, with a decrease in the number of bacteria in JP from the original 164 species to 35 species (Figure 2A). Species-level annotation of the microbiota in the environment, jiuqu, and pit mud revealed that the diversity of the GJ, GW, and JN bacterial communities was high, with 225, 219, and 187 species, respectively. Shared species among environmental, jiuqu, and pit mud samples were investigated (Figure 2A). A total of 164 species were detected in DS, of which 100 were detected in GJ, 93 in DH, 130 in GW, 115 in FQ, and 61 in the pit mud. At the time of the cellar release, the shared bacterial species in GJ, GW, FQ, and JN samples decreased (Figure 2A). For jiuqu, pit mud, and environmental samples, GW and FQ, GW and GJ, and JN and GJ, had shared a large number of bacterial species (Figure 2A). The microbial communities of fermented grains, jiuqu, pit mud, and the environment were analyzed by principal coordinate analysis (PCoA) (*R* > 0.98, *p* = 0.001) (Figure 2C). At the bacterial microbial community species level, GJ, DH, GW, JN, and JP each possessed their own unique bacterial microbial community structure, while DS and FQ shared a similar bacterial microbial community structure (Figure 2C).

There was no significant increase in the number of fungal species in DS and JP, with fungal species ranging from 31 to 40 species (Figure 2B). Species-level annotation of the microbiota in the environment, jiuqu, and pit mud revealed a high abundance of fungal communities in GJ and GW with 133 and 81 species, respectively (Figure 2B). Shared species among environmental, jiuqu, and pit mud samples were investigated. Thirty-six species were detected in DS, of which 30 were detected in GJ, 19 in DH, 20 in GW, 29 in FQ, and 26 in JN. At the time of cellar release, the shared fungal species in GJ, GW, FQ, and JN samples decreased (Figure 2B). For jiuqu, pit sludge, and environmental samples, GW and GJ, as well as GW and FQ had relatively more shared fungal species (Figure 2B). The microbial communities of fermented grains, jiuqu, pit mud, and the environment were analyzed by principal coordinate analysis (PCoA) (*R* > 0.98, *p* = 0.001) (Figure 2D). At the species level of the fungal microbial community, DH, GW, JN, DS, and JP each possessed their own unique bacterial microbial community structure. In contrast, FQ and GJ possessed similar fungal microbial community structure (Figure 2D).

### 3.2 Microbial community composition of fermented grains, before and after cellaring

**Figure 3:**
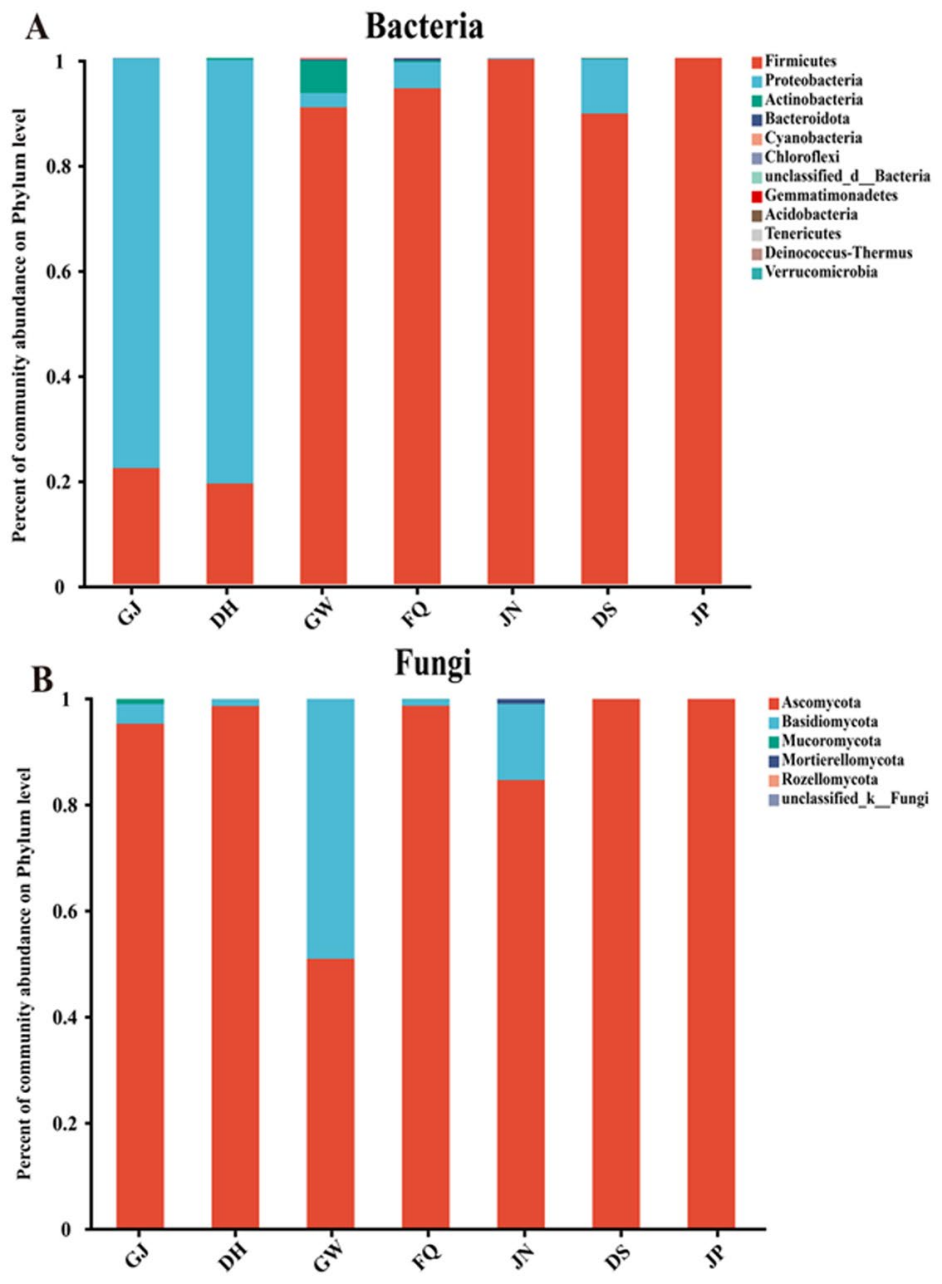
Microbial community structure in the environment, jiuqu, pit mud and fermented grains of Furou-type Baijiu before and after cellaring. Overview of bacterial taxa at the phylum level **(A)**; overview of fungal taxa at the phylum level **(B)**.

A study of the microbiota structure of fermented grains, before and after cellaring, revealed a total of 12 bacterial phyla and 6 fungal phyla in terms of dominant microorganisms at the phylum level (Figures 3A,B). For the bacterial phyla in GJ, DH, GW, FQ and JN samples, Proteobacteria acted as the dominant phylum in GJ and DH samples (mean relative abundance >77.8%), in GW and FQ, Firmicutes dominated (mean relative abundance >90.6%) and Proteobacteria acted as the subdominant phylum (mean relative abundance >2%) (Figure 3A). While actinobacteria had a higher relative abundance in GW than in other samples (average relative abundance >6.1%), Firmicutes dominated in JN samples (average relative abundance >99.7%) (Figure 3A). The dominant phylum of fermented grain bacteria, before and after cellaring, Firmicutes (mean relative abundance greater than 89.4%), Proteobacteria were sub-dominant in DS samples on average (mean relative abundance greater than 10%) were less dominant in JP samples (mean relative abundance less than 1%) (Figure 3A).

The Ascomycota phylum was the dominant phylum among all samples at the fungal phylum level, with sub-Ascomycota dominating in the GJ, DH, GW, FQ, and JN samples (mean relative abundance greater than 50%), Basidiomycota sub-dominating in the GW samples (mean relative abundance greater than 49%), and sub-dominating in the GJ, DH, FQ, and JN samples (mean relative abundance >1%) (Figure 3B). The dominant phylum of fermenting grain bacteria, before and after cellaring, is Ascomycota (mean relative abundance >99.99%) (Figure 3B).

**Figure 4:**
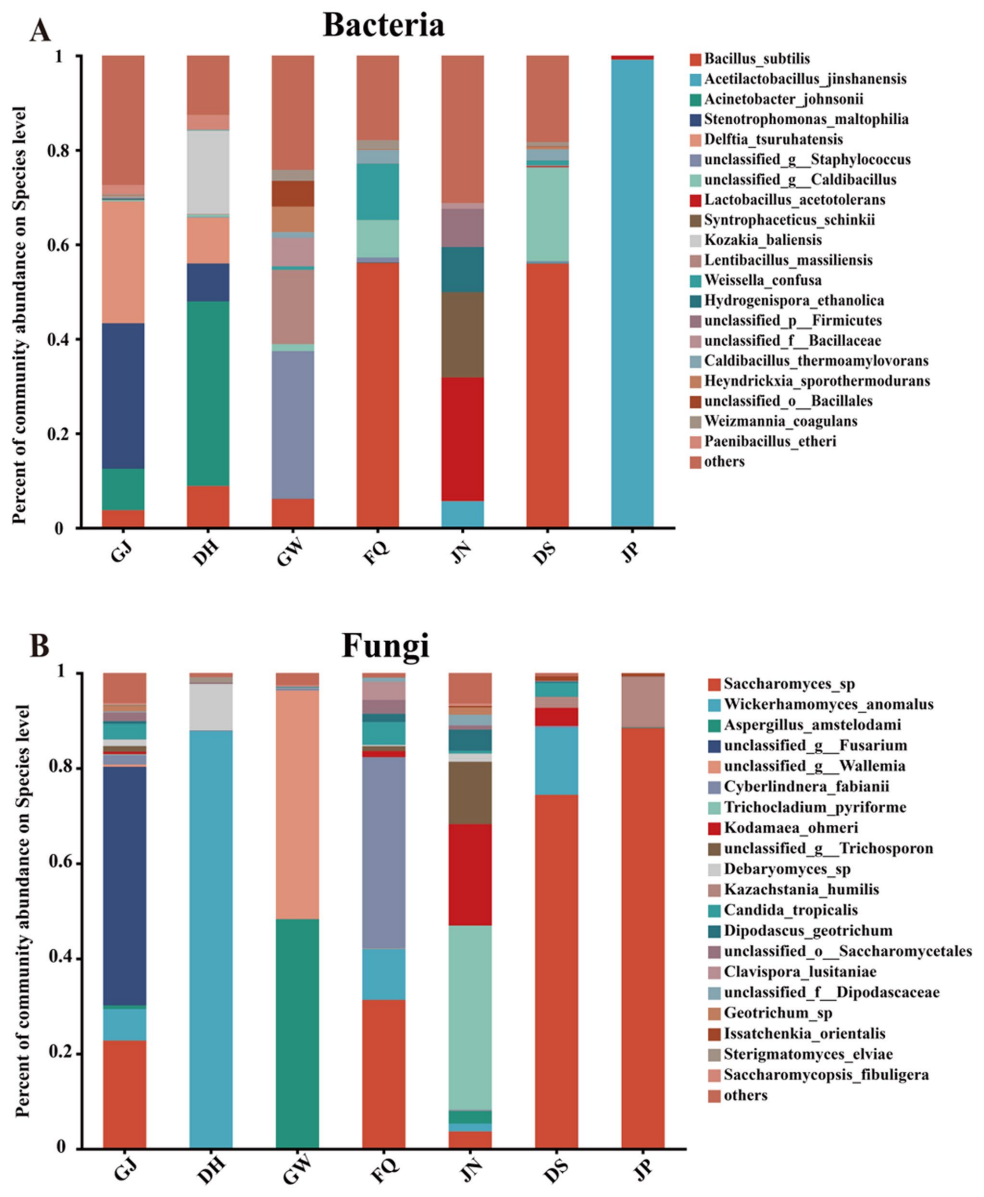
Microbial community structure in the environment, jiuqu, pit muck and fermented grains before and after cellaring of Furou-type Baijiu. Overview of bacterial taxa at the species level **(A)**; overview of fungal taxa at the species level **(B)**.

In terms of species-level dominant microorganisms (top 20 in terms of mean abundance ranking), *Acinetobacter johnsonii*, *Stenotrophomonas maltophilia*, and *Delftia tsuruhatensis* shared the dominant species in GJ and DH samples in GJ, DH, GW, FQ, and JN samples (mean relative abundance greater than 8%) (Figure 4A). Unclassified g _*Staphylococcus* and *Lentibacillus massiliensis* were dominant in GW (mean relative abundance greater than 15%) and less abundant in other samples (mean relative abundance less than 1%). *Bacillus subtilis* was a shared species in GJ, DH, GW, and FQ, but was most abundant in FQ (mean relative abundance >56%) (Figure 4A). *Lactobacillus acetotolerans*, *Syntrophaceticus schinkii*, *Hydrogenispora_ethanolica*, and unclassified p *Firmicutes* were dominant in the JN samples (average relative abundance greater than 8%) and low in the other samples (average relative abundance less than 1%; they were less abundant in different samples; mean relative abundance less than 1%). For DS and JP, *B. subtilis* and unclassified g *Caldibacillus* were the main dominant species in DS (average relative abundance greater than 19%), and the relative abundance of *Acetilactobacillus jinshanensis* gradually increased with the accumulation of fermented grains, and *A. jinshanensis* was dominant in JP (average relative abundance >98%) (Figure 4A).

In environmental, jiuqu and pit mud samples, *Wickerhamomyces anomalus* dominated in DH (mean relative abundance >87%) and was less abundant in JN, FQ and GJ (mean relative abundance >1%); *Aspergillus amstelodami*, unclassified g *Wallemia* dominated in GW, with lower abundance levels in the other samples (mean relative abundance less than 3%); *Cyberlindnera fabianii* dominated in FQ (mean relative abundance greater than 40%), *Trichocladium pyriforme* and *Kodamaea ohmeri* dominated in JN dominated (average relative abundance >21%) (Figure 4B). For DS and JP, *Saccharomyces* sp. dominated (mean relative abundance >74%). At the same time, it was sub-dominant in the GJ, FQ, and JN samples, with an 11.03% increase in *Saccharomyces* sp., before and after cellaring (mean relative abundance >88%). *W. anomalus* was higher in DH and lower in DS (mean relative abundance >14%), and *W. anomalus* became lower (mean relative abundance <1%) after cellar entry (Figure 4B).

### 3.3 GC–MS analysis results of volatile flavor substances before and after cellar entry

To investigate the flavor composition of fermented grains, before and after cellaring, volatile flavor compounds were detected in fermented grains, before and after cellaring, by gas chromatography–mass spectrometry (GC–MS). A total of 44 volatile compounds were detected in the GW, JN, DS, and JP samples, including 15 acids, 8 alcohols, 4 aldehydes, and 8 esters (Table 1). The relative flavor contents of palmitic acid, phenethyl alcohol, 3-methyl-1-butanol, and ethyl acetate were significantly increased (*p* < 0.05) after cellaring. Significantly enhanced (*p* < 0.05) (Table 1). Among them, 18 and 32 flavor substances were identified in GW and JP, respectively, and the relative contents of flavor substances in JP were significantly higher than those in DS (*p* < 0.05) (Table 1). Before and after cellaring, the relative contents of butyric acid, ricinoleic acid, and phenethyl alcohol were significantly increased, but the relative contents of methyl arachidate and methyl oleate were significantly decreased (*p* < 0.05) (Table 1).

**Table 1:** Determination of volatile flavor content during the fermented grains stacking process.

Name of substance	Mass concentration/(mg/kg)			
	GW	JN	DS	JP
Palmitic acid	6.62 ± 0.05b	9.27 ± 1.54b	–	36.18 ± 2.21a
Stearic acid	–	7.71 ± 1.57a	–	1.70 ± 0.71b
Butyric acid	–	80.79 ± 18.00a	1.13 ± 0.34b	13.12 ± 2.81b
2-Methylbutyric acid	17.09 ± 2.19b	20.70 ± 0.18a	–	15.88 ± 0.92b
Ricinoleic acid	–	43.59 ± 2.39a	0.54 ± 0.13b	2.65 ± 0.50b
1-Hexanoic acid	–	149.60 ± 12.93a	–	2.53 ± 0.20b
Acetic acid	2.00 ± 0.05b	–	–	10.55 ± 0.60a
Phenylacetic acid	2.35 ± 0.73b	4.11 ± 0.23a	–	–
3-Methylbutanoic acid	1.92 ± 0.10b	5.08 ± 1.36a	–	–
Pentanoic acid	–	80.34 ± 13.51a	–	–
n-Heptanoic acid	–	1.94 ± 0.07a	–	–
Octanoic acid	–	25.97 ± 2.38a	–	–
4-Methylcaprylic acid	–	1.73 ± 0.50a	–	–
Isobutyric acid	–	4.34 ± 0.18a	–	–
2-Methylundecanoic acid	–	–	24.13 ± 5.80a	–
Phenethyl alcohol	0.42 ± 0.03b	5.46 ± 1.39a	5.96 ± 1.38a	8.21 ± 0.43a
Palmitoleyl alcohol	3.27 ± 1.45a	4.41 ± 2.43a	0.73 ± 0.13a	1.58 ± 0.23a
3-Methyl-1-butanol	–	1.63 ± 0.56b	3.77 ± 1.07a	4.51 ± 0.50a
2,3-Butanediol	–	1.13 ± 0.69a	–	1.73 ± 0.22a
1-Butanol	–	3.19 ± 1.11a	–	1.81 ± 0.18a
2-Butanol	–	–	–	1.36 ± 0.15a
2-Furanmethanol	–	–	–	1.10 ± 0.05a
1-Hexanol	–	5.48 ± 0.28a	–	–
Ethyl octanoate	–	2.16 ± 0.14a	1.37 ± 0.03b	1.43 ± 0.24b
Methyl arachidate	–	–	11.31 ± 2.81a	2.04 ± 0.50b
Methyl stearate	–	–	–	1.38 ± 0.28a
Methyl oleate	3.48 ± 0.53a	1.49 ± 0.49b	4.16 ± 0.13a	1.58 ± 0.08b
Ethyl acetate	–	7.60 ± 2.56b	1.45 ± 0.23bc	26.22 ± 3.71a
Isopropyl palmitate	–	–	0.72 ± 0.44ab	1.86 ± 0.97a
Ethyl lactate	–	167.86 ± 187.25a	–	11.95 ± 0.62a
*Trans*-2-Hexenyl hexanoate	–	1.09 ± 0.09b	1.72 ± 0.87b	50.10 ± 2.48a
Mesitylene	3.20 ± 0.24b	–	–	4.51 ± 0.50a
All-*trans*-retinal	–	–	1.54 ± 0.30b	2.40 ± 0.30a
2-Ethyltoluene	–	–	0.91 ± 0.2b	1.22 ± 0.05a
3-Ethyltoluene	1.02 ± 0.17b	1.40 ± 0.18b	0.86 ± 0.10b	2.50 ± 0.34a
4-Ethyltoluene	–	–	–	2.16 ± 0.14a
o-Phthalaldehyde	–	–	1.04 ± 0.38a	1.45 ± 0.10a
Isolongifolene epoxide	–	–	0.69 ± 0.11b	3.26 ± 0.63a
2,6-Di-*tert*-butyl-p-benzoquinone	2.53 ± 0.11a	1.83 ± 0.03b	–	2.37 ± 0.05a
2,4-Di-*t*-butylphenol	11.23 ± 1.47b	13.01 ± 1.07b	–	17.81 ± 0.06a
4-*tert*-Butylphenol	–	–	–	2.87 ± 0.01a
3-Phenylpropanal	1.28 ± 0.39a	–	–	–
Vanillin	1.82 ± 0.03a	–	–	–
Benzoylcyclohexane	1.33 ± 0.23a	–	1.09 ± 0.65a	–

Different letters in the same row indicate significant differences (*p* < 0.05) and “–” indicates that the substance was not detected.

### 3.4 Correlation between volatile flavorants and major microbial species in fermented grains, before and after cellaring

**Figure 5:**
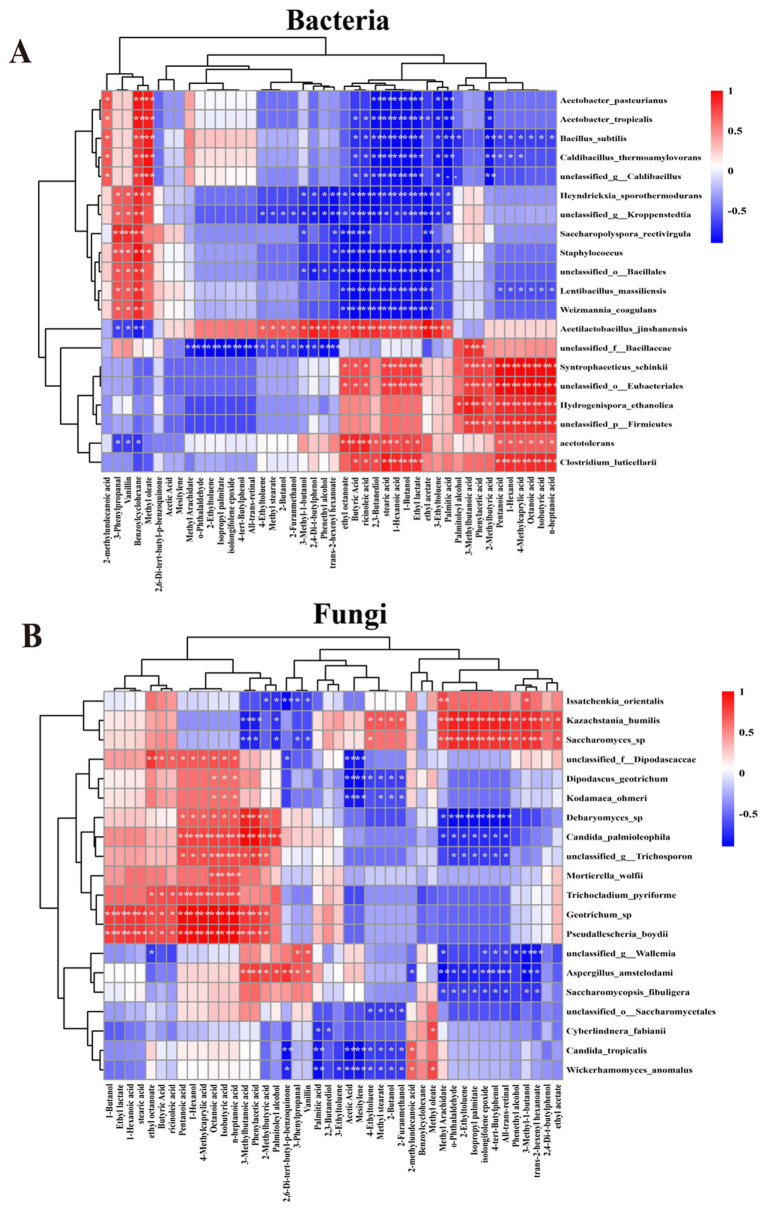
Heatmap of correlation analysis of major bacterial species **(A)** and fungal species **(B)** with volatile flavor substances based on Spearman’s correlation analysis [* indicates significant difference (*p* < 0.05); ** indicates highly significant difference (*p* < 0.01)].

To investigate the contributing role between volatile flavor compounds and major microbial species in fermented grains, before and after cellaring, bacteria and fungi that were dominant microorganisms (top 20 in average abundance ranking) at the species level were selected for this study, and Spearman’s correlation coefficients between relative abundance of the dominant microorganisms and the concentration of volatile compounds were further computed, which were visualized using heatmaps. The results showed that these microorganisms played a crucial role in the formation of volatile compounds (Figures 5A,B). Among them, *S. schinkii* and unclassified o *Eubacteriales* were positively correlated (*p* < 0.01) with five acids, including palmitic acid, octanoic acid, 4-methylcaprylic acid, and 1-hexanol (Figure 5A). *H. ethanolica*, unclassified p *Firmicutes*, and *C. luticellarii* were positively correlated (*p* < 0.01) with five esters and 1-hexanol. *A. jinshanensis* was correlated (*p* < 0.01) with four acids, such as stearic acid, butyric acid, and ricinoleic acid; five alcohols, such as 2,3-butanediol, phenethyl alcohol, and 1-butanol; three esters, such as ethyl octanoate and ethyl lactate, were positively correlated (*p* < 0.01) (Figure 5A). Additionally, *Acetobacter pasteurianus*, *Acetobacter tropicalis*, and *B. subtilis* showed positively correlation (*p* < 0.01) with the benzoylcyclohexane. Furthermore, methyl oleate was positively correlated (*p* < 0.01) with 2,3-butanediol and stearic acid, while 1-hexanoic acid and others were negatively correlated (*p* < 0.01). *Heyndrickxia sporothermodurans*, unclassified g *Kroppenstedtia*, *Saccharopolyspora rectivirgula*, etc. were associated with 3-phenylpropanal, vanillin, benzoylcyclohexane, which were positively correlated (*p* < 0.05) with ethyl octanoate, butyric acid, cricinoleic acid, etc., which were negatively correlated (*p* < 0.01) (Figure 5A). *Kazachstania humilis Saccharomyces* sp. were positively correlated (*p* < 0.01) with four esters and two alcohols (Figure 5B); *Geotrichum* sp. and *P. boydii* were positively correlated (*p* < 0.01) with six acids and hexanols (Figure 5B); *Candida tropicalis*, *W. anomalus*, *C. fabianii*, and unclassified o *Saccharomycetales* were negatively correlated with almost all substances.

### 3.5 Environmental contribution of fermented grain microbial communities

**Figure 6:**
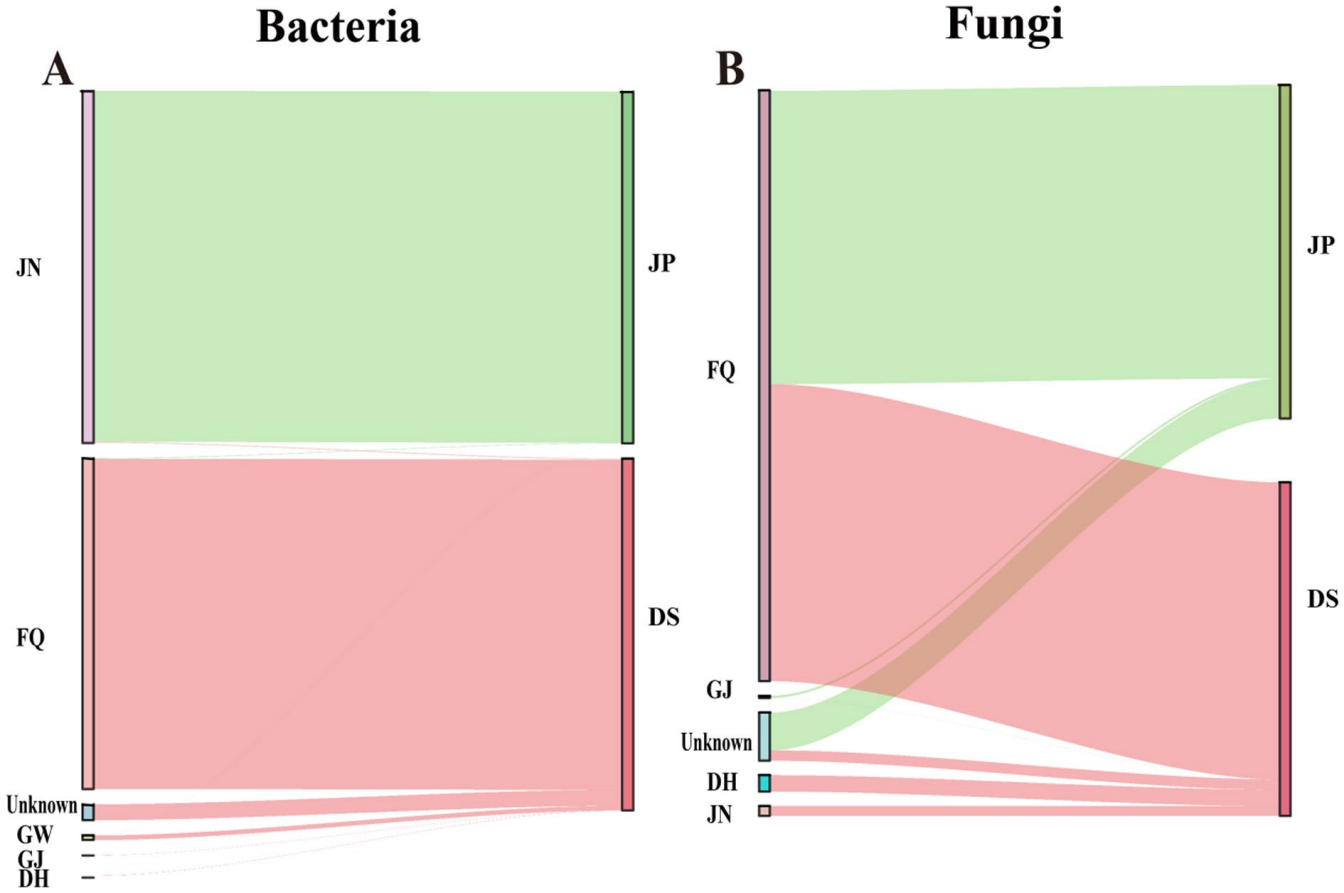
Source tracker microbial source estimation: **(A)** Source tracking analysis of bacterial communities in fermented grains before and after cellar entry of Furou-type Baijiu. **(B)** Source tracker analysis of fungal community of fermented grains before and after Furou-type Baijiu entry.

**Figure 7:**
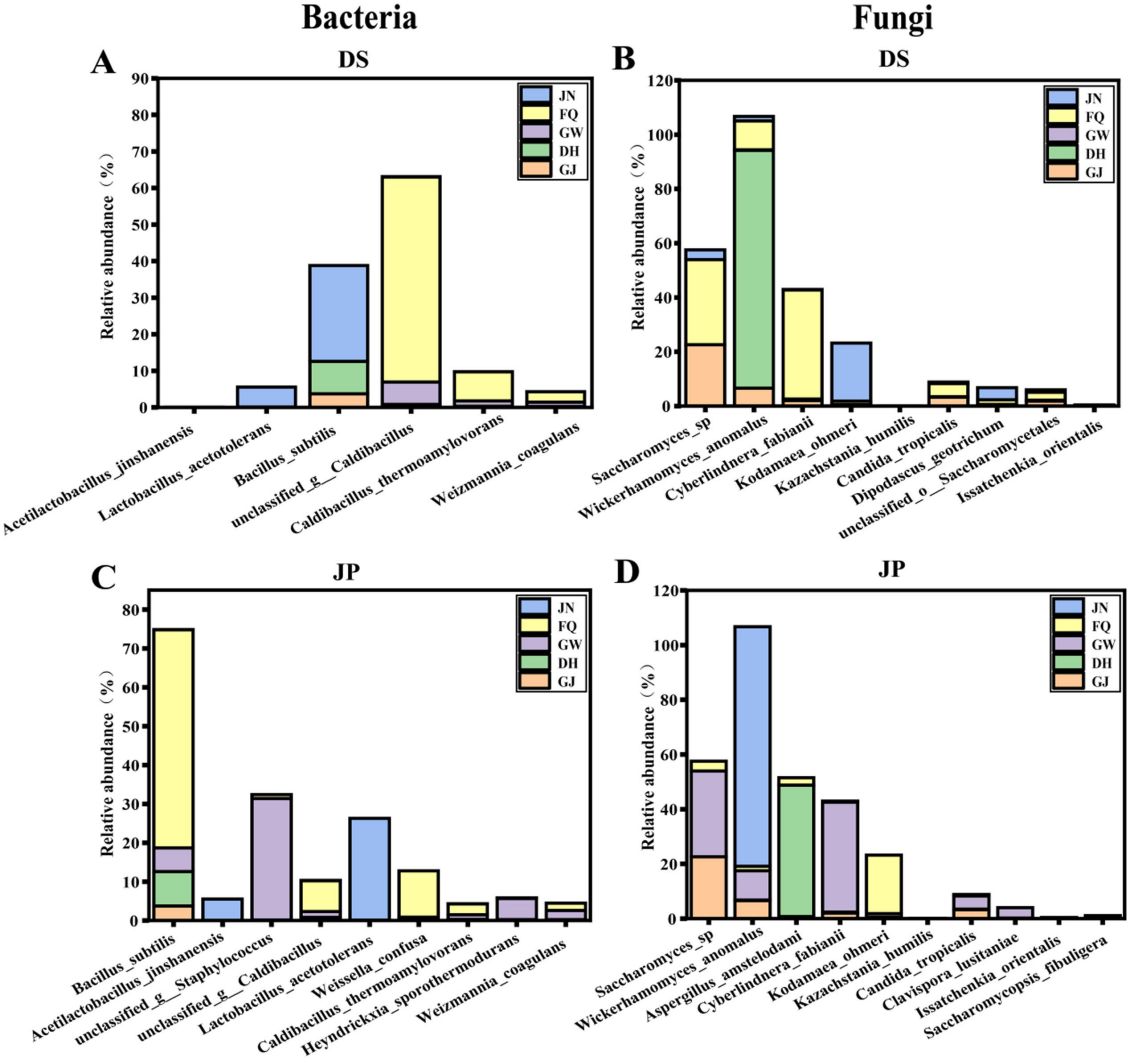
Bacterial species source analysis before and after 40 h of stacking **(A)** and fermentation of grains **(B)** in the cellar. Fungal species source analysis before and after 40 h stacking **(C)** and fermented grains **(D)** in the cellar.

SourceTracker was used to track the source of bacteria in fermented grains during Furou-type Baijiu fermentation. The results showed that at the species level, the main microbial source of bacteria in DS was FQ, and the main microbial source of bacteria in JP was JN (Figure 6A). FQ served as the main microbial source of DS (Figure 6A), where the microorganisms (with relative abundance >10%) were mainly *B. subtilis*, *Weissella_confusa*, unclassified g *Caldibacillus*, *Caldibacillus thermoamylovorans*, *Weizmannia coagulans*, and unclassified g *Staphylococcus* (Figure 7C). The main contributors to the bacterial community in JP were mainly from JN (Figure 6A). During fermentation, among the microorganisms (relative abundance >10%), the main species were *L. acetotolerans* and *A. jinshanensis* (Figure 7A). FQ was the main contributor to the fungal community, before and after cellaring (Figure 6B). FQ acted as the main source of microorganisms in DS (Figure 6B), in which microorganisms (relative abundance >10%) were mainly *C. fabianii*, *Saccharomyces* sp., *W. anomalus*, *K. ohmeri*, *C. tropicalis*, and *Clavispora lusitaniae* (Figure 7C). FQ is the main contributor to the fungal community in JP (Figure 6B). Among the microorganisms (relative abundance >10%), the main ones were *C. fabianii*, *Saccharomyces* sp., *W. anomalus*, *C. tropicalis*, unclassified o *Saccharomycetales*, *Dipodascus geotrichum*, and *K. ohmeri* (Figure 7D).

## 4 Discussion

In this study, we investigated the influence of environmental microorganisms on the microbial community of fermented grains and the role of microorganisms in the flavor formation of Furou-type Baijiu. The microbial community structure in fermented grains, jiuqu, and the environment (including tools) was resolved using the SMRT technique in combination with SourceTracker analysis. The volatile flavor compounds in the spirits before and after the cellar entry were detected by GC–MS. The volatile flavor substances were detected in the fermented grains, before and after cellaring. The results showed that microorganisms in the fermented grains and the environment migrated both artificially and naturally, enriching the diversity of the microbial community and promoting the production of flavor substances (Wang et al., 2017; Zhang et al., 2020a). The abundance of fungal and bacterial communities in JP was significantly lower than that in DS (*p* < 0.05) (Figures 1A–D). The characteristics of bacterial and fungal communities of fermented grains changed significantly, before and after cellaring (*p* < 0.05) (Figures 2A,B), which generally aligns with previous studies on the pattern of change in the abundance of microbial communities before and after cellaring for other aroma types of Baijiu (Duan et al., 2023; Lu et al., 2022; Yang et al., 2023; Zhang et al., 2023). At the gate level, a total of 12 bacterial phyla and 6 fungal phyla were detected, before and after cellaring. The dominant phyla Firmicutes, Ascomycota, and Proteobacteria (average relative abundance >10%) (Figures 3A,B) were the same as the results of previous studies on the dominant microorganisms at the gate level in the fermented grain stacks of other aroma-type Baijiu. The ascospores phyla were the main fungal phyla in the fermented grain stacks of saucy, strong and clear aroma-type The phylum Ascomycota was the main fungal phylum in the fermented grains of Baijiu, indicating that this phylum is a key fungal group in Chinese Baijiu brewing (Luo et al., 2022; Xue et al., 2023). At the species level, the microbial species richness of fermented grains changed significantly, both before and after cellaring. Compared with other aroma types, the bacterial species of Furou-type Baijiu before and after cellar entry were dominated by *B. subtilis*, unclassified g *Caldibacillus*, and *A. jinshanensis*, and the fungal species were dominated by *Saccharomyces* sp., *W. anomalus*, and *K. ohmeri* were the main fungal species. Similar to other aroma-type Baijiu stacking periods, but with a higher abundance of Furou-type Baijiu species, these microorganisms could produce large amounts of amylases, proteases, and hydrolytic enzymes that drove the saccharification and esterification of the fermented grains (Han et al., 2023; Hao et al., 2021; Ji et al., 2023; Li et al., 2016). *A. jinshanensis* increased and accounted for the largest proportion after entering the cellar. *A. jinshanensis*, as a new organic acid-induced growth-type bacterium, has been reported in the literature that they directly affect the production of lactic acid and ethanol during the fermentation process (Chen et al., 2025) and is active in the medium-chain fatty acid synthesis pathway, which is probably the most important metabolic pathway for medium-chain fatty acid esters. It is active in the middle-chain fatty acid synthesis pathway. It may be an important metabolizing microorganism for middle-chain fatty acid esters, which forms the typical characteristic of soy sauce-type Baijiu, that is, “empty glass fragrance” (Chen et al., 2024). This is also in line with the characteristics of Furou-type Baijiu and the fusion of four types of Baijiu aromas: strong, sauce-flavor, clear, and sesame. Fungal species were dominated by *Saccharomyces_*sp., *W. anomalus*, and *K. ohmeri* before entering the cellar. *Saccharomyces* sp. has high gene expression levels during fermentation, and *Saccharomyces* sp. promotes the efficient conversion of glucose to ethanol through the glycolytic pathway, where each glucose and fructose molecule is split and converted to ethanol, carbon dioxide, and a large number of volatile metabolites (Chen et al., 2022), which enhances the fermented grains’ wine-producing capacity, which has a positive effect on the development of Baijiu quality (Hu et al., 2019). and *W. anomalus* had a relatively low fermentation efficiency, but produced the most volatile compounds (Chen et al., 2022; Lu et al., 2022); The percentage of *Saccharomyces* sp. *gradually* increased and accounted for the largest percentage after cellar entry (Figures 4A,B). The results of the study are in agreement with previous studies, confirming the dominance of *Pichia* and *Saccharomyces* in the fungal community of fermented grains (Lina et al., 2022).

The core microbiota during fermented grain stacking was an important driver, thus indirectly affecting the flavor profile. A total of 44 volatile compounds were detected in GW, JN, DS, and JP samples. Eighteen and 32 flavor substances were identified in DS and JP, respectively, and the relative contents of flavor substances in JP were significantly higher than those in DS (*p* < 0.05) (Table 1). The relative contents of acids and alcohols were significantly elevated (*p* < 0.05) before and after the fermented grains were in the cellar, and the esterification reaction of acids and alcohols led to the production of new volatile organic compounds (Table 1). Ester compounds are components of the Baijiu aroma skeleton, giving Baijiu its unique flavor and style (Li et al., 2019).

SourceTracker identified that the bacterial communities of fermented grains were mainly sourced from JN and FQ, while the bacterial strains of DS were predominantly sourced from FQ, including *B. subtilis*, *W. confusa*, unclassified g *Caldibacillus*, *C. thermoamylovorans*, etc. The dominant bacterial species of DS, *B. subtilis* and unclassified g *Caldibacillus*, were also supplied by the FQ (Figure 6C), which not only provide saccharification and liquefaction capacity (Fan et al., 2018; Li et al., 2019) but also participate in a variety of flavors such as ester substances synthesis (Figure 5A). Among them, *B. subtilis*, which was not found in JP and JN in anaerobic environments for the time being, was the most abundant species in aerobic fermentation, and was enriched in the stacked fermentation stage in agreement with previous studies (Chen et al., 2024; Zhang et al., 2020b). The bacterial species of JP were mainly derived from JN, such as *L. acetotolerans* and *A. jinshanensis*, and the dominant bacterial species of JP, *A. jinshanensis*, underwent an increase in relative abundance after cellaring and accounted for the maximum, lower in GW, FQ, and DH. Still, relative abundance >5% in JN samples (Figures 4A,B, 6A,B) suggests that environmental microorganisms drove the increase in the relative abundance of these species in fermented grains. *L. acetotolerans* was found in fermented grains of clear, strong, and Sauce-flavor Baijiu *L. acetotolerans* is abundant and is considered to be the main functional genus during Baijiu fermentation that provides the fruity aroma and mellowness of Baijiu through the production of esterases and lipases for the synthesis of lactic acid, acetic acid, ethyl lactate, ethyl acetate, and other flavor-related compounds (Pang et al., 2021; Wang et al., 2016). *A. jinshanensis* is the main functional bacterium for vinegar production, which is more competitive under acidic conditions (Wang et al., 2023). *A. jinshanensis* is the key active bacterial species in the Baijiu fermentation process and is widely distributed in anaerobic and high-acid fermentation systems, which explains the abundance of this type of bacterium in the fermented grains compared to other samples (Liao et al., 2023). It has been shown that *A. jinshanensis* is the dominant species in Moutai flavor Baijiu, with a relative abundance of 92%, a finding that suggests it may contribute to the quality of Baijiu (Chen et al., 2025). Correlation analysis with volatile flavors revealed that *A. jinshanensis* was closely related to four acids, five alcohols, and three esters, and that *A. jinshanensis* affects core alcohol-producing microorganisms by regulating acidity, thereby indirectly regulating the expression of flavor components, such as ethanol fermentation. The concentrations of several acids, including octanoic acid and n-heptanoic acid, and 1-hexanol were correlated with the concentrations of *Geotrichum* sp., *P. boydii*, *S. schinkii*, and unclassified o *Eubacteriales* that were positively correlated with their relative abundance (Figure 5A). These microorganisms are mainly derived from pit mud and play an important role in the production of flavor substances. They can continuously migrate from the pit mud to the fermented grains during the fermentation process (Lu et al., 2022).

In Baijiu fermentation, fungi not only provide liquefaction, saccharification, and ethanol production for fermentation, but are also closely associated with the production of a wide range of flavors (Fan et al., 2018). SourceTracker tracking found that the fungal communities of fermented grains, before and after cellaring, mainly originated from the FQ, with *Saccharomyces* sp. as the main DS, and JP was also contributed by dominant fungi, which were also contributed by FQ as the primary contributor. Spearman correlation analysis of microorganisms and volatile flavor substances revealed that *Saccharomyces* sp. was closely associated with four esters and two alcohols, which could continuously migrate from pit mud to fermented grains during fermentation, and the increase in abundance after stacking also improved the Baijiu production capacity of fermented grains (Lu et al., 2022).

## 5 Conclusion

In this study, the effects of Daqu and its environmental microorganisms on the microbial community structure of fermented grains before and after cellar entry of Furou-type Baijiu were investigated by combining the SMRT technique, and volatile flavor substances were analyzed by GC–MS. Microorganisms in the environment, before and after cellaring, significantly increased the diversity of microbial communities in fermented grains, through anthropogenic and natural migration, and promoted the production of flavor substances. The concentrations of several acids, such as octanoic acid, isobutyric acid, n-heptanoic acid, and 1-hexanol were positively correlated with the microorganisms in the pit mud. Ester compounds are the key components of Baijiu flavor, and the esterification reaction between alcohols and acids generates new volatile organic compounds. The bacterial communities in DS and JP originated from FQ and JN, respectively. The fungal communities in fermented grains, before and after cellaring, primarily originated from FQ. These microorganisms migrated into the fermented grains continuously during fermentation, affecting the volatile flavor substances of some acids and alcohols. The microbial community changes before and after the fermented grains were cellared were crucial for the formation of Baijiu styles. These results enhance our understanding of the microbial sources and their relationship with flavor substances in Furou-type Baijiu, and provide an important theoretical basis for improving the quality and production process of Baijiu.

## Data Availability

The datasets presented in this study can be found in online repositories. The names of the repository/repositories and accession number(s) can be found in the article/Supplementary material.

## References

[ref1] Boyang X. Shanshan X. Jing C. Wei S. Dongdong M. Xuefeng W. . (2022). Analysis of the microbial community and the metabolic profile in medium-temperature Daqu after inoculation with *Bacillus licheniformis* and *Bacillus velezensis*. LWT Food Sci. Technol. 160:113214. doi: 10.1016/j.lwt.2022.113214

[ref2] Chen L. Li F. Yang F. Chen B. Du H. Wang L. . (2024). Microbiome dynamics and environment driving factors throughout pit fermentation of Jiang-flavor baijiu: a multi-omics study. Food Biosci. 60:104363. doi: 10.1016/j.fbio.2024.104363, 40553273

[ref3] Chen X. Wu Y. Zhu H. Wang H. Lu H. Zhang C. . (2022). Turning over fermented grains elevating heap temperature and driving microbial community succession during the heap fermentation of sauce-flavor baijiu. LWT 172:114173. doi: 10.1016/j.lwt.2022.114173

[ref4] Chen L. Zheng H. Cheng K. Li C. Qin X. Wang G. . (2025). Deciphering the acidophilia and acid resistance in *Acetilactobacillus jinshanensis* dominating baijiu fermentation through multi-omics analysis. Food Microbiol. 125:104655. doi: 10.1016/j.fm.2024.104655, 39448165

[ref5] Duan Z. Wu Y. Zhang C. Niu J. Zhao J. Li W. . (2023). Comparison of fungal communities and flavour substances in surface and inner layers of fermented grains during stacking fermentation of sauce-flavour baijiu. J. Biosci. Bioeng. 136, 295–303. doi: 10.1016/j.jbiosc.2023.06.010, 37544799

[ref300] Edgar R. C. (2013). UPARSE: highly accurate OTU sequences from microbial amplicon reads. Nat. Methods 10, 996–998., 23955772 10.1038/nmeth.2604

[ref6] Fan G. Sun B. Fu Z. Xia Y. Huang M. Xu C. . (2018). Analysis of physicochemical indices, volatile flavor components, and microbial community of a light-flavor Daqu. J. Am. Soc. Brew. Chem. 76, 209–218. doi: 10.1080/03610470.2018.1424402

[ref7] Gou M. Wang H. Yuan H. Zhang W. Tang Y. Kida K. (2015). Characterization of the microbial community in three types of fermentation starters used for Chinese liquor production. J. Inst. Brew. 121, 620–627. doi: 10.1002/jib.272

[ref8] Guiming F. Mengfei D. Kedan C. Yanru C. Wenqin C. Choufei W. . (2021). Peak-temperature effects of starter culture (Daqu) on microbial community succession and volatile substances in solid-state fermentation (Jiupei) during traditional Chinese special-flavour baijiu production. LWT Food Sci. Technol. 152:112132. doi: 10.1016/j.lwt.2021.112132

[ref9] Han P.-J. Luo L.-J. Han Y. Song L. Zhen P. Han D.-Y. . (2023). Microbial community affects Daqu quality and the production of ethanol and flavor compounds in baijiu fermentation. Food Secur. 12:2936. doi: 10.3390/foods12152936, 37569205 PMC10418397

[ref10] Hao F. Tan Y. Lv X. Chen L. Yang F. Wang H. . (2021). Microbial community succession and its environment driving factors during initial fermentation of Maotai-flavor baijiu. Front. Microbiol. 12:669201. doi: 10.3389/fmicb.2021.669201, 34025626 PMC8139626

[ref11] Hestand M. S. Ameur A. (2019). The versatility of SMRT sequencing. Genes 10:24. doi: 10.3390/genes10010024, 30621217 PMC6357146

[ref12] Hu Y. Lei X. Zhang X. Guan T. Wang L. Zhang Z. . (2021). Characteristics of the microbial community in the production of Chinese rice-flavor baijiu and comparisons with the microflora of other flavors of baijiu. Front. Microbiol. 12:673670. doi: 10.3389/fmicb.2021.673670, 33995338 PMC8116502

[ref13] Hu A. Nie Y. Yu G. Han C. He J. He N. . (2019). Diurnal temperature variation and plants drive latitudinal patterns in seasonal dynamics of soil microbial community. Front. Microbiol. 10:674. doi: 10.3389/fmicb.2019.00674, 31001239 PMC6454054

[ref14] Ji X. Zhang L. Yu X. Chen F. Guo F. Wu Q. . (2023). Selection of initial microbial community for the alcoholic fermentation of sesame flavor-type baijiu. Food Res. Int. 172:113141. doi: 10.1016/j.foodres.2023.113141, 37689904

[ref15] Jin G. Zhu Y. Xu Y. (2017). Mystery behind Chinese liquor fermentation. Trends Food Sci. Technol. 63, 18–28. doi: 10.1016/j.tifs.2017.02.016

[ref16] Li W. Fan G. Fu Z. Wang W. Xu Y. Teng C. . (2019). Effects of fortification of Daqu with various yeasts on microbial community structure and flavor metabolism. Food Res. Int. 129:108837. doi: 10.1016/j.foodres.2019.108837 32036879

[ref17] Li P. Lin W. Liu X. Wang X. Gan X. Luo L. . (2016). Effect of bioaugmented inoculation on microbiota dynamics during solid-state fermentation of Daqu starter using autochthonous of *Bacillus*, *Pediococcus*, *Wickerhamomyces* and *Saccharomycopsis*. Food Microbiol. 61, 83–92. doi: 10.1016/j.fm.2016.09.004 27697173

[ref18] Li H. Liu S. Liu Y. Hui M. Pan C. (2023). Functional microorganisms in baijiu Daqu: research progress and fortification strategy for application. Front. Microbiol. 14:1119675. doi: 10.3389/fmicb.2023.1119675, 36778882 PMC9911690

[ref19] Liao W. Li Y. Zhang Y. Yang Y. Yang T. Miao L. (2023). Comparative analysis of the transcriptional responses of *Acetilactobacillus jinshanensis* BJ01 to organic acids. Arch. Microbiol. 205:381. doi: 10.1007/s00203-023-03715-5, 37968407

[ref20] Lina Z. Ya W. Jiale X. Shaobin G. Ying W. Xuan L. . (2022). Distinct succession of abundant and rare fungi in fermented grains during Chinese strong-flavor liquor fermentation. LWT Food Sci. Technol. 163:113502. doi: 10.1016/j.lwt.2022.113502

[ref21] Liu M.-K. Tang Y.-M. Guo X.-J. Zhao K. Tian X.-H. Liu Y. . (2017). Deep sequencing reveals high bacterial diversity and phylogenetic novelty in pit mud from Luzhou Laojiao cellars for Chinese strong-flavor baijiu. Food Res. Int. 102, 68–76. doi: 10.1016/j.foodres.2017.09.075, 29196000

[ref22] Lu Z.-M. Wang Z.-M. Zhang X.-J. Mao J. Shi J.-S. Xu Z.-H. (2017). Microbial ecology of cereal vinegar fermentation: insights for driving the ecosystem function. Curr. Opin. Biotechnol. 49, 88–93. doi: 10.1016/j.copbio.2017.07.006 28843369

[ref23] Lu Y. Zhang C. Zhao H. Min W. Zhu H. Wang H. . (2022). Effect of environmental microorganisms on fermentation microbial community of sauce-flavor *baijiu*. Food Secur. 12:10. doi: 10.3390/foods12010010, 36613226 PMC9818559

[ref24] Luo A. Yang N. Yang J. Hao J. Zhao J. Shi S. . (2022). Effects of microbial interspecies relationships and physicochemical parameters on volatile flavors in sorghum-based fermented grains during the fermentation of Shanxi light-flavored liquor. Food Sci. Nutr. 11, 1452–1462. doi: 10.1002/fsn3.3185, 36911827 PMC10002873

[ref25] Pang X.-N. Chen C. Huang X.-N. Yan Y.-Z. Chen J.-Y. Han B.-Z. (2021). Influence of indigenous lactic acid bacteria on the volatile flavor profile of light-flavor baijiu. LWT 147:111540. doi: 10.1016/j.lwt.2021.111540

[ref26] Pang X.-N. Han B.-Z. Huang X.-N. Zhang X. Hou L.-F. Cao M. . (2018). Effect of the environment microbiota on the flavour of light-flavour baijiu during spontaneous fermentation. Sci. Rep. 8:3396. doi: 10.1038/s41598-018-21814-y, 29467508 PMC5821866

[ref27] Shi S. Zhang L. Wu Z.-y. Zhang W.-x. Deng Y. Zhong F.-d. . (2011). Analysis of the fungi community in multiple- and single-grains Zaopei from a Luzhou-flavor liquor distillery in western China. World J. Microbiol. Biotechnol. 27, 1869–1874. doi: 10.1007/s11274-010-0645-7

[ref500] Stackebrandt E. Goebel B. M. (1994). Taxonomic note: a place for DNA-DNA reassociation and 16S rRNA sequence analysis in the present species definition in bacteriology. Int. J. Systematic Evolutionary Microbiol. 44, 846–849.

[ref28] Tan Y. Zhong H. Zhao D. Du H. Xu Y. (2019). Succession rate of microbial community causes flavor difference in strong-aroma baijiu making process. Int. J. Food Microbiol. 311:108350. doi: 10.1016/j.ijfoodmicro.2019.108350, 31614280

[ref29] Tong W. Li Y. Yang Y. Huang Z. Wang S. Huang D. . (2023). Dynamic analysis caffeic acid production driven by the key physicochemical factor and microbial community succession in baijiu Daqu: a multi-microorganism fermentation of solid-state fermentation system. LWT 190:115542. doi: 10.1016/j.lwt.2023.115542

[ref30] Wang L. (2022). Research trends in Jiang-flavor baijiu fermentation: from fermentation microecology to environmental ecology. J. Food Sci. 87, 1362–1374. doi: 10.1111/1750-3841.16092, 35275413

[ref31] Wang X. Du H. Zhang Y. Xu Y. (2017). Environmental microbiota drives microbial succession and metabolic profiles during Chinese liquor fermentation. Appl. Environ. Microbiol. 84, e02369–17. doi: 10.1128/aem.02369-17 PMC5795089 29196296

[ref32] Wang X. Hu K. Liu F. Mou J. Lai J. Zhang M. . (2023). Isolation and characterization of a gas-producing and acid-resistant bacterium from spoiled vinegar. Int. J. Food Microbiol. 394:110167. doi: 10.1016/j.ijfoodmicro.2023.110167, 36913840

[ref33] Wang Q. Zhang H. Liu X. (2016). Microbial community composition associated with Maotai liquor fermentation. J. Food Sci. 81, M1485–M1494. doi: 10.1111/1750-3841.13319, 27122124

[ref34] Wei Y. Zou W. Shen C. H. Yang J. G. (2020). Basic flavor types and component characteristics of Chinese traditional liquors: a review. J. Food Sci. 85, 4096–4107. doi: 10.1111/1750-3841.15536, 33190291

[ref35] Xu Y. Sun B. Fan G. Teng C. Xiong K. Zhu Y. . (2017). The brewing process and microbial diversity of strong flavour Chinese spirits: a review. J. Inst. Brew. 123, 5–12. doi: 10.1002/jib.404

[ref36] Xue T.-d. Zhang J.-h. Wang T.-r. Bai B.-q. Hou Z.-x. Cheng J.-f. . (2023). Reveal the microbial communities and functional prediction during the fermentation of fen-flavor baijiu via metagenome combining amplicon sequencing. Ann. Microbiol. 73:16. doi: 10.1186/s13213-023-01719-6

[ref37] Yang L. Xian C. Li P. Wang X. Song D. Zhao L. . (2023). The spatio-temporal diversity and succession of microbial community and its environment driving factors during stacking fermentation of Maotai-flavor baijiu. Food Res. Int. 169:112892. doi: 10.1016/j.foodres.2023.112892, 37254340

[ref38] You L. Zhao D. Zhou R. Tan Y. Wang T. Zheng J. (2021). Distribution and function of dominant yeast species in the fermentation of strong-flavor baijiu. World J. Microbiol. Biotechnol. 37:26. doi: 10.1007/s11274-020-02988-y, 33427975

[ref39] Zhang H. Wang L. Tan Y. Wang H. Yang F. Chen L. . (2020a). Effect of *Pichia* on shaping the fermentation microbial community of sauce-flavor baijiu. Int. J. Food Microbiol. 336:108898. doi: 10.1016/j.ijfoodmicro.2020.108898 33129005

[ref40] Zhang Y. Xu C. Xing G. Yan Z. Chen Y. (2023). Evaluation of microbial communities of Chinese Feng-flavor Daqu with effects of environmental factors using traceability analysis. Sci. Rep. 13:7657. doi: 10.1038/s41598-023-34506-z, 37169808 PMC10175296

[ref41] Zhang H. Zhang L. Yu X. Xu Y. (2020b). The biosynthesis mechanism involving 2,3-Pentanedione and Aminoacetone describes the production of 2-Ethyl-3,5-dimethylpyrazine and 2-Ethyl-3,6-dimethylpyrazine by *Bacillus subtilis*. J. Agric. Food Chem. 68, 3558–3567. doi: 10.1021/acs.jafc.9b07809, 32065523

