# Supplementary material for: Microbial community and flavor analyses of fermented grains of Furou-type Baijiu
Source: Front Microbiol. 2025 Dec 8;16:1628609. doi: 10.3389/fmicb.2025.1628609 (PMC12722865; doi:10.3389/fmicb.2025.1628609)
Supplement: Supplementary file 1 [file Table_1.docx]

| Category | Region | Samples | Sequences Bases(bp) | Sequences Bases(bp) |
| --- | --- | --- | --- | --- |
| Bacteria | 21 | 967466 | 1425416216 | 1473 |
| Fungi | 21 | 801098 | 520509060 | 649 |

Table S1 Number of valid sequences and range of read lengths for environmental, jiuqu, pit mud and fermented grains


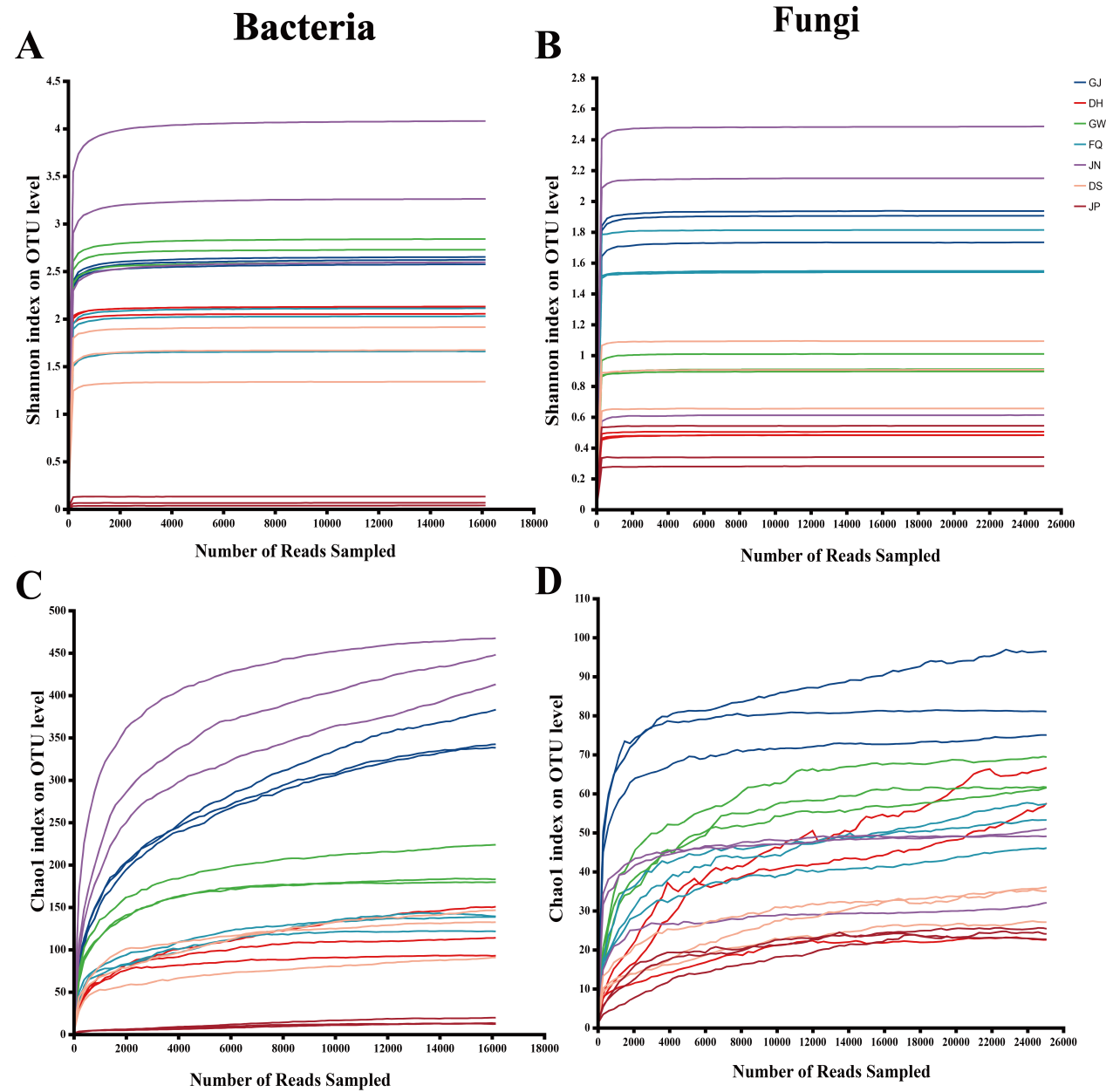


Fig. S1 Dilution curves of Shannon index (A, C) and Chao1 index (B, D) for different samples.
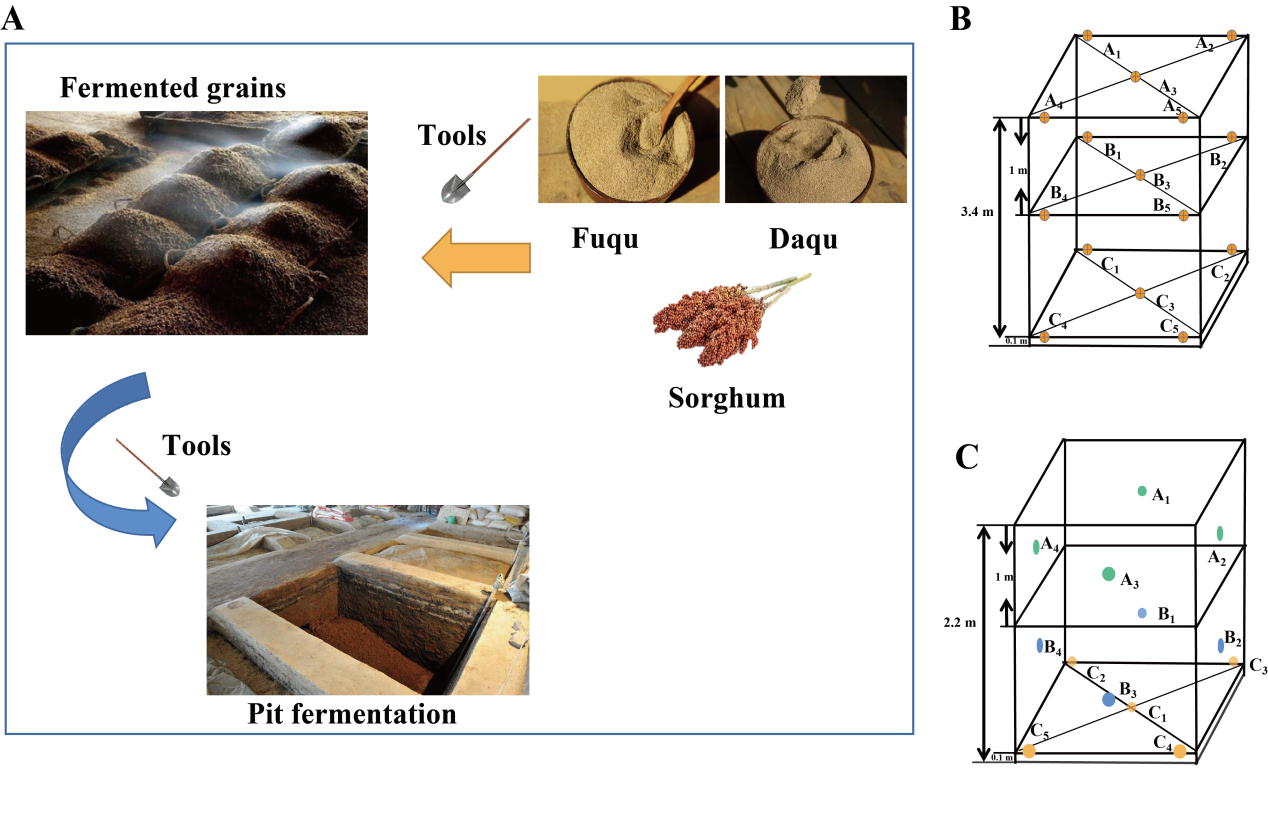


Figure S2 Furou-type baijiu production model (A). Sampling location sampling of Daqu, fuqu (B) and pit mud (C)
